# Oral Contraceptives Modulate the Relationship Between Resting Brain Activity, Amygdala Connectivity and Emotion Recognition – A Resting State fMRI Study

**DOI:** 10.3389/fnbeh.2022.775796

**Published:** 2022-03-14

**Authors:** Shanice Menting-Henry, Esmeralda Hidalgo-Lopez, Markus Aichhorn, Martin Kronbichler, Hubert Kerschbaum, Belinda Pletzer

**Affiliations:** ^1^Center for Cognitive Neuroscience, University of Salzburg, Salzburg, Austria; ^2^Department of Psychology, University of Salzburg, Salzburg, Austria; ^3^Neuroscience Institute, Paracelsus Medical University, Salzburg, Austria; ^4^Department of Biosciences, University of Salzburg, Salzburg, Austria

**Keywords:** emotion recognition, amygdala, limbic system, resting state fMRI, brain connectivity, ALFF, hormonal contraceptives, progestins

## Abstract

Recent research into the effects of hormonal contraceptives on emotion processing and brain function suggests that hormonal contraceptive users show (a) reduced accuracy in recognizing emotions compared to naturally cycling women, and (b) alterations in amygdala volume and connectivity at rest. To date, these observations have not been linked, although the amygdala has certainly been identified as core region activated during emotion recognition. To assess, whether volume, oscillatory activity and connectivity of emotion-related brain areas at rest are predictive of participant’s ability to recognize facial emotional expressions, 72 participants (20 men, 20 naturally cycling women, 16 users of androgenic contraceptives, 16 users of anti-androgenic contraceptives) completed a brain structural and resting state fMRI scan, as well as an emotion recognition task. Our results showed that resting brain characteristics did not mediate oral contraceptive effects on emotion recognition performance. However, sex and oral contraceptive use emerged as a moderator of brain-behavior associations. Sex differences did emerge in the prediction of emotion recognition performance by the left amygdala amplitude of low frequency oscillations (ALFF) for anger, as well as left and right amygdala connectivity for fear. Anti-androgenic oral contraceptive users (OC) users stood out in that they showed strong brain-behavior associations, usually in the opposite direction as naturally cycling women, while androgenic OC-users showed a pattern similar to, but weaker, than naturally cycling women. This result suggests that amygdala ALFF and connectivity have predictive values for facial emotion recognition. The importance of the different connections depends heavily on sex hormones and oral contraceptive use.

## Introduction

Emotion recognition, specifically the recognition of facial expressions, is central to social interaction (Mannava, 2012) and important for both the ontogenetic and phylogenetic development of our species (Schmidt and Cohn, 2001; Shenk et al., 2013). It is also selectively impaired in a variety of disorders associated with poor social functioning, e.g., autism or schizophrenia (Kohler et al., 2003; Kuusikko et al., 2009; Comparelli et al., 2013; Uljarevic and Hamilton, 2013). We are now looking back at centuries of extensive research on emotion recognition dating back to Darwin’s seminal work Darwin (1989). The recognition of at least five basic emotional expressions identified by Ekman (1992)—happiness, sadness, anger, fear, disgust—is considered universal across cultures (Elfenbein and Ambady, 2002; Scherer et al., 2011; but see Russell, 1994; and Barrett et al., 2019).

Neuroimaging studies demonstrate that emotion recognition is associated with the activation of limbic regions including amygdala, basal ganglia, hippocampus, parahippocampus, anterior cingulate cortex, and the orbitofrontal cortex (Lane et al., 1998; Bush et al., 2000; Adolphs, 2002; Gur et al., 2002; Streit et al., 2003; Lancaster et al., 2019). The response to facial emotions by these limbic regions is modulated by the relevance of the emotional content (Gur et al., 2002). The amygdala in particular seems to be a key structure in the recognition of emotions and consistently activates during the processing of facial emotion expressions, especially during fearful expressions (Thomas et al., 2001; Adolphs, 2002; Gur et al., 2002; Hariri et al., 2002; Derntl et al., 2008b). This group of nuclei, functionally connected to extensive subcortical and cortical regions (Roy et al., 2009), has been the focus of a multitude of neuroimaging studies describing how the brain responds to recognizing different emotions in various contexts. Subcortical connections of the amygdala allow the processing of subliminal or unconscious facial emotion expressions, while cortical connections are involved in the conscious recognition of facial emotion expressions (Adolphs, 2002). Bilateral amygdala activity is stronger when there is explicit emotion recognition, and thus conscious emotion processing (Gur et al., 2002; Habel et al., 2007). The activation of the amygdala in response to fear and anger is lateralized with stronger reactivity in the right hemisphere, and decreases after habituation (Thomas et al., 2001; Hariri et al., 2002).

Previous research indicates that emotion recognition and amygdala reactivity and connectivity are modulated by sex and participant’s hormonal status (Engman et al., 2016). In most studies, women display a higher accuracy in recognizing facial emotions, particularly negative emotions, than men (Thayer and Johnsen, 2000; Montagne et al., 2005; Hoffmann et al., 2010; Rukavina et al., 2018; Connolly et al., 2019; Olderbak et al., 2019). Limbic areas respond with stronger activation to emotional expressions in men compared to women (Weisenbach et al., 2014) and amygdala reactivity to emotional expressions is more lateralized in men (Thomas et al., 2001).

However, these sex differences are further modulated by women’s hormonal status, i.e., their menstrual cycle phase or hormonal contraceptive use. An average menstrual cycle lasts 29 days, divided into follicular and luteal phase (Fehring et al., 2006). Ovarian hormone levels are lowest at the beginning of each cycle, i.e., during menses (Abraham et al., 1972). During the follicular phase estrogen levels rise and peak right before ovulation, while progesterone levels remain low. The consecutive luteal phase is characterized by high progesterone levels and medium estradiol levels. Androgen levels also vary over the menstrual cycle, with lower levels of testosterone at the beginning and end of the menstrual cycle (Judd and Yen, 1973). These fluctuations in hormonal levels are not seen in women using combined oral contraceptives (COCs). COCs contain a synthetic estrogen, mostly ethinylestradiol, and a synthetic progestin (Pletzer and Kerschbaum, 2014). These synthetic steroids downregulate the hypothalamic–pituitary–gonadal axis and decrease the production of endogenous sex hormones, including testosterone (Wiegratz et al., 2003). Therefore, COC users show reduced and stable levels of endogenous ovarian hormones over time (Fleischman et al., 2010), most comparable with levels seen during menses in naturally cycling women. However, the synthetic steroids show strong estrogenic and progestogenic activity due to their high binding affinities to the estrogen and progesterone receptors, respectively (Sitruk-Ware, 2008; Stanczyk et al., 2013). Accordingly, it is hard to discern, whether the effects of COCs on emotion recognition are attributable to the reduction of endogenous hormones or the estrogenic and progestogenic actions of the synthetic hormones.

Ovarian hormonal fluctuations along the menstrual cycle have been related to emotion recognition and associated brain activation. During the follicular phase there is a higher emotion recognition accuracy compared to the luteal phase (Derntl et al., 2008a). On the contrary, it appears that women are more sensitive to facial cues signaling nearby threats during the luteal phase, when progesterone levels are high (Conway et al., 2007). A neuroimaging review of Toffoletto et al. (2014) showed that emotional processing leads to different activation across the distinct cycle phases in the amygdala, medial prefrontal cortex, orbitofrontal cortex, dorsolateral prefrontal cortex and inferior frontal gyrus. The most consistent finding in fMRI studies is that the amygdala has a stronger response to negative emotional stimuli during the luteal phase (Sundström Poromaa and Gingnell, 2014). The influence of ovarian hormones on emotion recognition and concurrent amygdala activation in naturally cycling women is not always found (Sundström Poromaa and Gingnell, 2014; Shirazi et al., 2020).

Hormonal contraceptive use also influences emotion recognition in women (Hamstra et al., 2014; Pahnke et al., 2019). Some studies suggest that women using COCs are less accurate in recognizing emotions (Hamstra et al., 2014; Pahnke et al., 2019), while other studies report no significant differences (Radke and Derntl, 2016; Shirazi et al., 2020). These inconsistencies between studies may arise from a lack of control for the type of COCs used. Apart from their progestogenic activity, progestins can be classified by their interaction with androgen receptors (Pletzer and Kerschbaum, 2014). Androgenic progestins are derived from 19-nortestosterone and act as agonists of the androgen receptor, whereas anti-androgenic progestins bind selectively to the progesterone receptor or act as antagonists of the androgen receptor (Sitruk-Ware, 2008). Accordingly, androgenic progestins have a more androgenic side effect profile than anti-androgenic progestins (Gurvich et al., 2020). Differential effects of androgenic and anti-androgenic progestins on brain structure have already been reported (Pletzer et al., 2015). To the best of our knowledge, Gurvich et al. (2020) are the only group that also examined the effect of oral contraceptive type on emotion recognition performance. They found an effect of androgenic vs. anti-androgenic oral contraceptive use on facial emotion recognition, in advantage of androgenic oral contraceptive users. Since men have higher androgen levels compared to women, their performance on facial emotion recognition is of interest to compare with women using androgenic and anti-androgenic COCs.

Pahnke et al. (2019) suggest that COCs impair the recognition of emotions *via* changes in the activity and connectivity in the prefrontal and temporal brain regions, caused by the reduction in endogenous hormone levels. It has indeed been observed that in hormonal contraceptive users, the amygdala shows not only reduced reactivity to emotional stimuli (Petersen and Cahill, 2015), but reduced gray matter volumes and altered connectivity to pre-frontal and central areas during the resting state (Lisofsky et al., 2016; Engman et al., 2018). However, to the best of our knowledge, these alterations in resting state connectivity patterns in hormonal contraceptive users has not been related to their ability to recognize facial emotional expressions. Despite the extensive research into the brain reactivity to emotional expressions, no study has so far assessed whether certain characteristics of the resting brain, like the size and functional connectivity of limbic areas such as the amygdala, are predictive of emotion recognition performance.

Understanding these brain-behavior associations and their modulation by androgenic vs. anti-androgenic oral contraceptives use is meaningful in the larger context of women’s mental health. Hormonal contraceptives are used by 150 million women worldwide (Petitti, 2003) and though their effects on the brain are not yet fully understood (Brønnick et al., 2020), they have been implicated in cognitive, emotional and social functioning (Montoya and Bos, 2017). Of particular interest with regards to mental health are their effects on emotion processing. Although long-term users of COC appear to experience stabilizing effects on mood (Oinonen and Mazmanian, 2002), about 4–10% of women report severe adverse mood effects (Sundström Poromaa and Segebladh, 2012). Accordingly, some studies report an increased risk of COC users to develop depression (e.g., Skovlund et al., 2016), while other studies suggest a protective effect of COCs regarding mood disorders (Cheslack-Postava et al., 2015). It is yet unclear, why these effects of COCs on mood appear to be bidirectional, but a differential responsiveness of the brain to synthetic steroids seems to be a plausible explanation. Accordingly, it is important to identify brain areas, which are (i) associated with emotional processing already at rest and (ii) modulated by COC use. Since the risk for adverse mood effects appears to be increased in adolescent compared to adult participants (de Wit et al., 2020) and with androgenic compared to anti-androgenic COCs (Sundström Poromaa and Segebladh, 2012), age and androgenicity of progestins appear to be important modulators in that respect.

The present manuscript focuses on identifying the neural bases of emotion recognition performance in the resting brain in relation to individual hormonal status. In order to do so, we assess the gray matter volumes and the resting state oscillatory activity in relation to emotion recognition performance. We also considered whether this association was modulated by sex and hormonal status. Furthermore, bilateral amygdalae were defined as regions of interest (ROIs) and its volume, oscillatory activity and functional connectivity at rest assessed in relation to the participant’s ability to recognize facial emotional expressions. We hypothesize that larger amygdalae, along with higher resting activity or connectivity of these areas are predictive of better emotion recognition performance. Taking into account the hormonal status of participants, we hypothesize that any behavioral differences in emotion recognition performance between different groups of hormonal contraceptive users (androgenic vs. anti-androgenic) and non-users can be explained by differences in the resting brain. In order to clearly characterize the differences between androgenic and anti-androgenic contraceptives, men are used as a comparison group.

## Materials and Methods

### Participants

Seventy-two healthy young participants (mean age: 25.34 ± 6.35 years). 20 men (mean age: 28.35 ± 8.83 years), 20 women with natural menstrual cycle (mean age: 25.95 ± 6.10 years) and 32 hormonal contraceptive users (mean age: 23.09 ± 3.25 years) took part in this study. The hormonal contraceptive group can be divided into 16 androgenic users (mean age: 24.56 ± 3.03 years) and 16 anti-androgenic users (mean age: 21.63 ± 2.83 years). All participants were white Caucasian and college students or employees at university. Naturally cycling women had a regular menstrual cycle with a mean duration of 29.08 days (SD = 1.56 days). Within the natural cycling (NC) group, only participants who had not been using any hormonal contraceptives or intrauterine device for the past 6 months were included. Of the 32 hormonal contraceptive users, 16 were taking older generation hormonal contraceptives containing androgenic progestins (Desogestrel, Levonorgestrel or Gestoden) and 16 were taking newer generation hormonal contraceptives containing anti-androgenic progestins (Drospirenone, Chlormadinone Acetate, Dienogest). Within the androgenic oral contraceptive (OC) and anti-androgenic OC group, participants needed be on their current OC for at least 6 months before start of the study. All participants gave their signed written consent to participate in the study. The study was approved by the University of Salzburg’s ethic committee and conforms to the Code of Ethics of the World Medical Association (Declaration of Helsinki). Participants had no psychological, endocrinological or neurological disorders and did not display any brain structural abnormalities on structural MRI.

### Behavioral Data Acquisition

**FIGURE 1:**
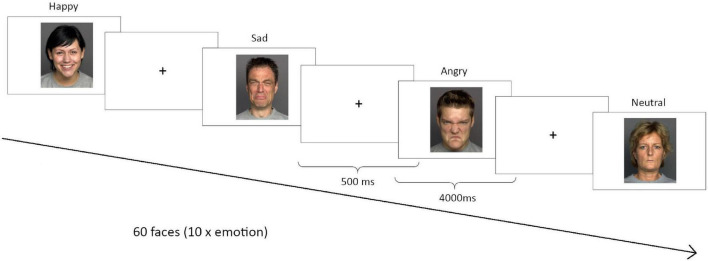
Emotion recognition task. Three different versions were performed in each correspondent session. During each session, participants viewed 60 faces from the FACES database (http://faces.mpib-berlin.mpg.de/), 10 for each emotion: anger, sadness, happiness, disgust, fear or neutral.

In order to assess a trait value of emotion recognition performance, participants completed three different versions of an emotion recognition task with approximately 10 days between the respective sessions. Performance measures were then averaged across the three test sessions. The order of versions across test sessions was counterbalanced. During each session, participants viewed 60 faces from the FACES database^1^ on a computer screen, 10 each displaying either a neutral expression or happiness, sadness, anger, fear or disgust (see Figure 1 for example faces). The order of emotions was randomized during each session. Participants had 4 s to rate the emotional expression of each face as neutral, happy, sad, angry, fearful or disgusted by pressing the, respectively, marked keys on a computer keyboard. Mean reaction times (RT) and accuracy were recorded for each emotion. The inter-stimulus-interval was 500 ms.

In order to control for potential hormonal influences on emotion recognition performance, hormonal status of the participating women was counterbalanced across test sessions. Naturally cycling women completed the three sessions during the following phases of their menstrual cycle: early follicular/menstrual phase (cycle days 1–5; low estradiol and progesterone), pre-ovulatory phase (1–3 days before ovulation; high estradiol), mid-luteal phase (4–10 days past ovulation; high estradiol and progesterone). Ovulation was assumed 14 days before the onset of the next period as expected by participants’ self-reports of cycle length and onset of the last period and was confirmed by commercial ovulation tests. The menses session on average took place on day 5 (SD = 2.92), the ovulation session on average took place on day 15 (SD = 2.34), the luteal session on average took place on day 23 (SD = 4.70). Cycle phases were confirmed by the assessment of the sex hormones from saliva samples using DeMediTec ELISA kits for estradiol, progesterone and testosterone, as reported in Pletzer et al. (2016). Two naturally cycling women were excluded due to a mismatch between the expected and the actual cycle phase. Hormonal contraceptive users completed one test session during the active pill phase (hormone containing pills) and one test session during the placebo phase (placebo pills or no pills). The third test session was scheduled randomly either in the active pill or placebo phase.

### Behavioral Analyses

In order to explore whether the hormonal status was predictive of emotion recognition performance we investigated menstrual cycle phase and oral contraceptive pill effects through linear mixed models in R version 3.6.1.^2^ (R Core Team, 2019) with *nlme* (Pinheiro et al., 2014) and *multcomp* (Hothorn et al., 2008) packages. Using *performance* as dependent variable and *participant number* (PNr) as random effect, for naturally cycling women *cycle phase* and *session number* were used as fixed effects (independent variables): e.g., RT ∼ 1|PNr + cycle phase + session. For pill users, *pill phase, pill type* and its *interactive effect* alongside the *session* number were used as the independent variables: e.g., RT ∼ 1|PNr + pill phase*pill type + session. We then address whether the groups (men, naturally cycling women, A-OC women and AA-OC women) differed from each other in emotion recognition performance using *group* and *session number* as fixed effects, and *participant number* as random effect: e.g., RT ∼ 1|PNr + group + session. In all the aforementioned cases, we accounted for multiple testing by first, FDR-correcting for the 5 emotions (anger, sadness, disgust, fear, happiness), and second, conducting Tukey-corrected all-pairwise comparisons between the different levels of the factors *cycle phase*, *session* and *group*.

### MRI Data Acquisition

Depending on the group participants were assigned to, they were scanned during one, two or all three test sessions, although only one scan is relevant to the current study. Of the 72 participants, two participants did not complete all planned scans and were therefore excluded from further analysis. Brain images from one male participant were not included due to bad quality, resulting in a final sample of 69 participants (19 men, 18 women with a natural menstrual cycle and 32 hormonal contraceptive users). Men were scanned during one visit only. Naturally cycling women were scanned during their menses, pre-ovulatory and luteal cycle phase. Women using oral contraceptives were scanned during pill intake and during pill pause.

However, changes in brain structure and resting brain activity related to cycle phase or pill phase were described elsewhere (Pletzer et al., 2015, 2016) and are not within the scope of the current manuscript. Therefore, only the menses scan from naturally cycling women, and the active pill scan from oral contraceptive users were used for the analyses. Given that oral contraceptives use downregulates the hypothalamic–pituitary–gonadal axis, decreasing the endogenous ovarian hormones production to levels comparable to those observed in naturally cycling women during menses, the menses session of naturally cycling women was chosen as the most comparable in terms of endogenous hormone levels.

Functional and high resolution structural images were acquired on a Siemens Magnetom TIM Trio 3 Tesla scanner (Siemens Healthcare) at the Christian Doppler Klinik (Salzburg, Austria). For resting state a T2-weighted gradient echo planar (EPI) sequence with 36 transversal slices orientated parallel to the AC-PC line (whole-brain coverage, TE = 30 ms, TR = 2,250 ms, flip angle 70°, slice thickness 3.0 mm, matrix 192 × 192, FOV 192 mm, in-plane resolution 2.6 mm × 2.6 mm). Participants were instructed to close their eyes, relax and let their mind flow. For structural images we acquired a T1-weigthed 3D MPRAGE sequence of 5 min 58 s (160 sagital slices, slice thickness = 1 mm, TE 291 ms, TR 2,300 ms, TI delay 900 ms, FA 9°, FOV 256 mm × 256 mm).

### MRI Data Analysis

#### Preprocessing of Structural Images

In order to analyze the structural data, we performed voxel-based morphometry (VBM) using the CAT12 toolbox^3^ of the SPM12 software^4^ (Ashburner and Friston, 2000). During this procedure, scans are corrected for bias-field inhomogeneities, spatially registered to an anatomical template, and each voxel is classified as gray matter, white matter or cerebrospinal fluid, accounting for partial volume effects. CAT12 segmentation was applied through default options, including affine registration to SPM12 tissue probability maps and European brain templates, light affine preprocessing and moderate (0.5) strength of local adaptive segmentation, skull stripping and final clean-up for segmentation. Spatial normalization to the MNI template was used to correct intra-subject bias, and non-linear normalization parameters to control for inter-subject variability (Luders et al., 2004). For smoothing, we used an 8 mm full width at half maximum Gaussian kernel.

#### Preprocessing of Functional Images

In order to pre-process the functional images, we first applied a 3d-despiking as implemented in AFNI^5^, and then the standard procedures and templates from SPM12 including realignment and unwarping of the functional images using the fieldmap, co-registration of the functional images to the segmented structural images, normalization of functional images using the parameters as estimated by CAT12, and spatial smoothing using a 6 mm kernel. Finally, we perform ICA-AROMA non-aggressive removal of artifactual components on the resulting images (Pruim et al., 2015).

#### Calculation of ALFF Maps

In order to assess how strongly the BOLD- signal fluctuates, and as a measure of spontaneous neuronal activity, the amplitude of low-frequency fluctuations (ALFF) was calculated using the DPABI toolbox (Yan et al., 2016). In order to do so, the signal was first filtered (bandpass, 0.01–0.08 Hz) to remove effects of very-low-frequency drift and high frequency noise as caused e.g., by respiratory and heart rhythms. Then, ALFF maps were calculated as the average square root of the power spectrum within this range of frequencies.

#### ROI Analyses

In a first step, we focused on the amygdala as a region of interest (ROI). Masks were constructed for the left and right amygdala *via* the wfu-pickatlas toolbox, using the Neuromorphometrics atlas. Gray matter (GM) volumes from the left and right amygdala were extracted using the get_totals script by G. Ridgeway^6^. ALFF in the left and right amygdala was extracted from a one-sample *t*-test over all subjects using eigenvalues. In order to address whether left and right amygdala volumes, as well as left and right amygdala ALFF were predictive of emotion recognition performance and whether this association was modulated by hormonal status, we ran linear models in R 3.6.1. Each emotion was explored using RT/Accuracy as dependent variable and GM volume/ALFF as well as its interaction with group as independent variable (e.g., RT ∼ GM*Group, Acc ∼ ALFF*Group). We accounted for multiple testing by FDR-correcting for the 5 emotions (anger, sadness, disgust, fear, happiness). If a significant interaction between performance and group was observed, correlation (Pearson’s *r*) of performance with GM/ALFF was calculated for each group.

#### Calculation of Seed-Based Connectivity Maps

In order to investigate the connectivity of each of the left and right amygdala to the rest of the brain, we assessed seed-to-voxel connectivity using the CONN-toolbox^7^ (Whitfield-Gabrieli and Nieto-Castanon, 2012). For each subject we calculated connectivity maps through standard procedures and templates, using 6 movement parameters as well as 5 white matter and cerebrospinal fluid components as regressors in a first-level analysis and a band-pass filter of 0.008 to 0.09 Hz.

#### Whole Brain Analyses

In a second step, we used SPM second level analyses at the whole brain level to assess whether emotion recognition performance related to gray matter volumes or ALFF outside the amygdala on the one hand, and whether emotion recognition performance related to amygdala connectivity on the other hand. To that end, and separately for each emotion, we performed full factorial models on modulated GM maps, ALFF maps and connectivity maps, using either emotion recognition RT or emotion recognition accuracy as regressors and modeling their interaction with group. If a significant interaction between performance and group was observed, brain parameters were extracted from significant clusters and their correlation (Pearson’s *r*) with performance explored for each group. We also explored whether accounting for age *via* partial correlations affected any correlation coefficients. However, due to the age-homogeneity of the sample, this was not the case. Accordingly, age was not considered further in the analyses. In order to account for multiple testing, the uncorrected *p*-value threshold was divided by the number of emotions (5) and therefore set to *p* = 0.0002. Results are reported when Family-Wise Error (FWE) corrected *p* < 0.05.

## Results

### Behavior

Within the naturally cycling group of women (NC), there was no cycle phase effect on either emotion recognition reaction time (RT) or emotion recognition accuracy for any of the emotions (all *F*_2,31_ < 2.15, *p*_FDR_ > 0.05. For women on oral contraceptives (OC) there were no significant effects of the pill phase (all *F*_1,46_ < 5.10, *p*_FDR_ > 0.05), the pill type (A or AA) (all *F*_1,27_ < 6.10, *p*_FDR_ > 0.05) or their interaction (all *F*_1,46_ < 4.40, *p*_FDR_ > 0.05) on either emotion recognition RT or emotion recognition accuracy. When considering the whole sample, there were no differences in performance (RT or accuracy) between the groups for any of the emotions (all *F*_3,65_ < 3.35, *p*_FDR_ > 0.05). Emotion recognition RT and accuracy on the different emotions for the four groups can be found in Tables 1, 2.

**TABLE 1:** Reaction time for emotion recognition.

Group	Session	Angry	Sad	Fear	Happy	Disgust
Men	1	1,960.68 (317.08)	1,993.27 (445.51)	2,328.84 (487.99)	1,303.05 (170.46)	2,073.35 (522.52)
	2	1,686.76 (370.36)*	1,875.31 (386.45)	2,078.09 (567.13)*	1,278.01 (268.55)	1,920.69 (480.46)*
	3	1,713.39 (282.43)*	1,952.4 (401.38)	2,021.63 (500.02)*	1,272.04 (167.5)	1,832.67 (434.06)*
NC women	1	1,677.46 (306.76)	1,846.7 (414.7)	1,991.86 (395.68)	1,229.62 (238.71)	1,820.05 (471.46)
	2	1,615.58 (221.99)*	1,890.15 (382.19)	1,993.44 (484.65)*	1,147.72 (211.22)	1,713.28 (391.88)*
	3	1,566.16 (348.84)*	1,873.44 (362.66)	1,955.08 (441.67)*	1,221.95 (205.67)	1,787.83 (308.96)*
A-OC women	1	1,709.23 (304.54)	1,900.21 (498.44)	2,010.89 (519.72)	1,203.37 (241.76)	1,671.62 (257.6)
	2	1,618.56 (300.64)*	1,810.91 (453.7)	1,858.49 (356.62)*	1,108.9 (219.32)	1,585.55 (298.76)*
	3	1,637.84 (384.47)*	1,773.26 348.54)	1,834.54 (447)*	1,152.74 (276.16)	1,622.3 (287.85)*
AA-OC women	1	1,834.08 (400.94)	1,982.83 (439.01)	2,255.06 (611.17)	1,244 (192.94)	1,887.47 (323.63)
	2	1,669.09 (237.88)*	1,989.74 449.24)	2,014.23 (390.7)*	1,207.06 (171.7)	1,817.52 (303.47)*
	3	1,733.11 (231.83)*	1,889.88 (349.86)	2,120.76 (446.16)*	1,175.14 (141.91)	1,761.33 (200.02)*

Mean in milliseconds (SD) *p < 0.05 for the second and third session compared to the first one.

**TABLE 2:** Accuracy for emotion recognition.

Group	Session	Angry	Sad	Fear	Happy	Disgust
Men	1	80.00 (17)	50.00 (24.49)	56.32 (27.53)	98.95 (3.15)	72.11 (17.18)
	2	87.89 (12.73)	64.21 (19.53)*	70.00 (30.55)	98.42 (5.01)	76.84 (21.1)
	3	88.95 (15.6)	52.63 (25.79)*	67.37 (25.79)	97.89 (4.19)	75.26 (20.1)
NC women	1	88.89 (12.78)	55.56 (19.77)	78.33 (14.65)	100 (0)	82.78 (14.47)
	2	89.44 (9.38)	66.67 (27.65)*	81.67 (22.03)	99.44 (2.36)	73.33 (18.15)
	3	87.06 (12.13)	61.76 (28.34)*	78.24 (28.34)	97.65 (5.62)	78.82 (18.67)
A-OC women	1	88.13 (14.71)	51.25 (21.25)	76.25 (22.17)	98.75 (3.42)	72.50 (22.06)
	2	90.67 (12.23)	63.33 (23.5)*	83.26 (14.44)	98.67 (3.52)	76.00 (21.31)
	3	92.67 (10.33)	67.67 (19.17)*	88.00 (15.68)	100 (0)	77.67 (16.57)
AA-OC women	1	89.38 (10.63)	63.75 (17.08)	78.75 (21.56)	98.13 (4.03)	78.75 (17.84)
	2	87.50 (14.38)	71.25 (16.28)*	72.50 (22.66)	98.13 (4.03)	86.25 (14.55)
	3	93.75 (9.75)	71.25 (20.62)*	80.00 (16.73)	98.75 (3.42)	87.12 (16.01)

Mean percentage hits (SD) *p < 0.05 for the second and third session compared to the first one.

The number of sessions had an effect on RT for anger (*F*_2,133_ = 7.78, *p*_FDR_ = 0.003), fear (*F*_2,133_ = 3.78, *p*_FDR_ = 0.04), and disgust (*F*_2,133_ = 4.70, *p*_FDR_ = 0.03). For these emotions participants were faster during the second and third session than the first session, irrespective of the group (SE < 0.15, |z| > 2.3, p_tukey_ < 0.05). The number of sessions also had an effect on the accuracy for sadness (*F*_2,133_ = 7.04, *p*_FDR_ = 0.006). Participants were more accurate during the second and third session than the first session, irrespective of the group (SE < 0.14, |z| > 2.5, p_tukey_ < 0.03).

### ROI-Based Analysis

In order to address, whether left and right amygdala volumes, as well as left and right amygdala ALFF were predictive of emotion recognition performance and whether this association was modulated by hormonal status, we ran linear models with *performance* as dependent variable and *GM volume/ALFF* as well as its interaction with *group* as fixed effects: e.g., RT ∼ GM*Group. Here, we also accounted for multiple testing by first, FDR-correcting for the 5 emotions (anger, sadness, disgust, fear or happiness), and second, conducting Tukey-corrected all-pairwise comparisons between the different levels of *group*.

#### Gray Matter Volumes

Neither left nor right amygdala volumes were predictive of either emotion recognition reaction time or emotion recognition accuracy (all *F*_3,61_ < 3.00, *p*_FDR_ > 0.05) and no interactions with group were observed (all *F*_3,61_ < 2.18, *p*_FDR_ > 0.05).

#### ALFF

**FIGURE 2:**
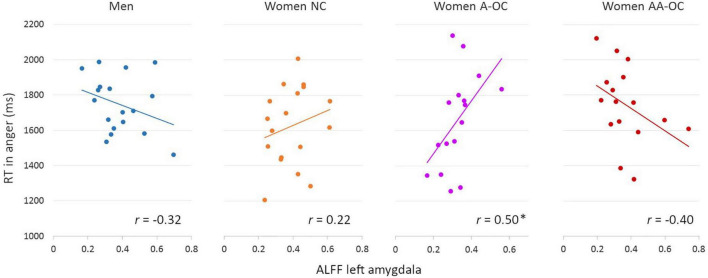
Relationship between the amplitude of low-frequency fluctuations (ALFF) in the left amygdala and reaction time (RT) for angry faces by group. ALFF and RT were found to be moderately positively correlated for women on A-OC. *p < 0.05.

The ALFF of the left amygdala showed a trend interactive effect with group on the RT for anger (*F*_3,60_ = 3.58, *p* = 0.018), however, it did not survive the multiple comparison correction (*p*_FDR_ = 0.09). ALFF and RT were found to be moderately positively correlated for women on A-OC (*r*_14_ = 0.50; Figure 2). The higher the ALFF in the left amygdala, the slower anger recognition in women using A-OC.

No further main effects or interaction with group was observed for left nor right amygdala ALFF on emotion recognition RT (all *F*_3,61_ < 3.58, *p*_FDR_ > 0.05).

Neither left nor right amygdala ALFF were predictive of emotion recognition accuracy and no interactions with group were observed (all *F*_3,60_ < 2.90, *p*_FDR_ > 0.05).

### Whole Brain Analyses

**FIGURE 3:**
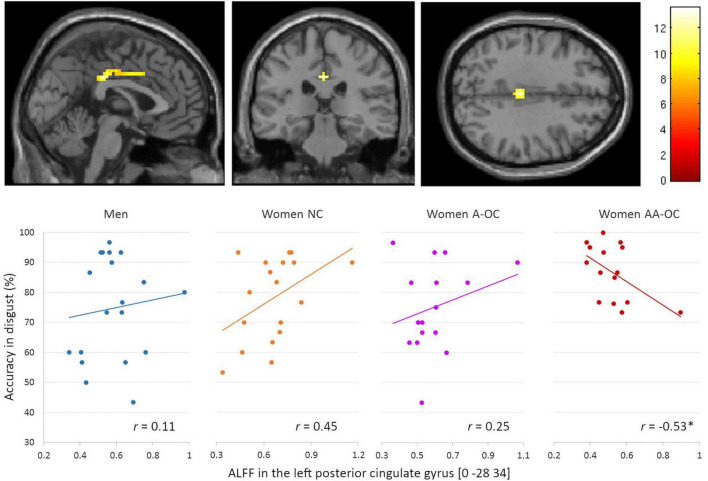
Relationship between the amplitude of low-frequency fluctuations (ALFF) in the left posterior cingulate gyrus and accuracy for disgust faces by group. ALFF and accuracy were found to be moderately negatively correlated for women on AA-OC. *p < 0.05.

No main effects of performance were observed for the gray matter volume or ALFF. No interaction of group by performance was found for the gray matter volume. Regarding the ALFF, we observed a significant group*accuracy interaction in the left posterior cingulate gyrus (PCC) [0 –28 34], *k* = 48 voxels, *F* = 13.59, p_FWE_ < 0.001, for the emotion of disgust. ALFF and accuracy were found to be moderately negatively correlated for women on AA-OC (*r*_14_ = −0.53; Figure 3). The lower the ALFF in the left PCC, the higher disgust recognition accuracy in women using AA-OC.

**FIGURE 4:**
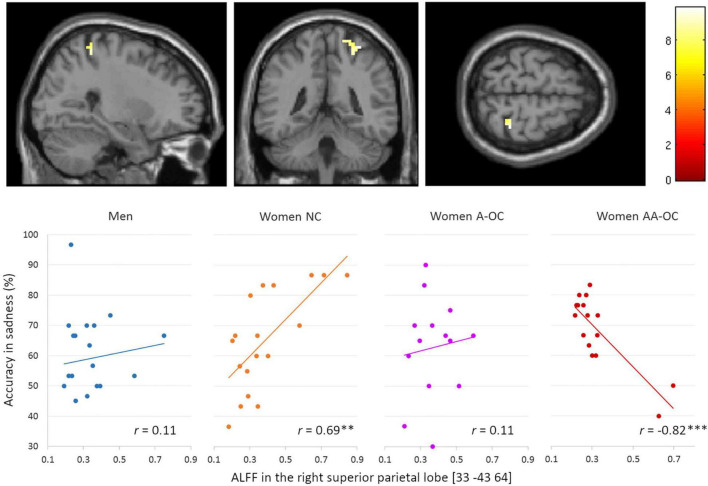
Relationship between the amplitude of low-frequency fluctuations (ALFF) in the in the right superior parietal lobe and accuracy of sadness by group. ALFF and accuracy were found to be positively correlated for naturally cycling women, whereas they were negatively correlated for women on AA-OC. **p < 0.01, ***p < 0.001.

We also observed a significant group*accuracy interaction for the ALFF in the right superior parietal lobe (SPL) [33 –43 64], *k* = 20 voxels, *F* = 4.09, p_FWE_ = 0.02, for the emotion of sadness. ALFF and accuracy were found to be positively correlated for naturally cycling women (*r*_16_ = 0.69) whereas it was negatively correlated for women on AA-OC (*r*_14_ = −0.82) (Figure 4).

The lower the ALFF in the right SPL, the lower sadness recognition accuracy in naturally cycling women, while the highest sadness recognition accuracy in women using AA-OC.

No interaction of group*RT of any emotion was observed for the ALFF.

#### Amygdala Connectivity

**FIGURE 5:**
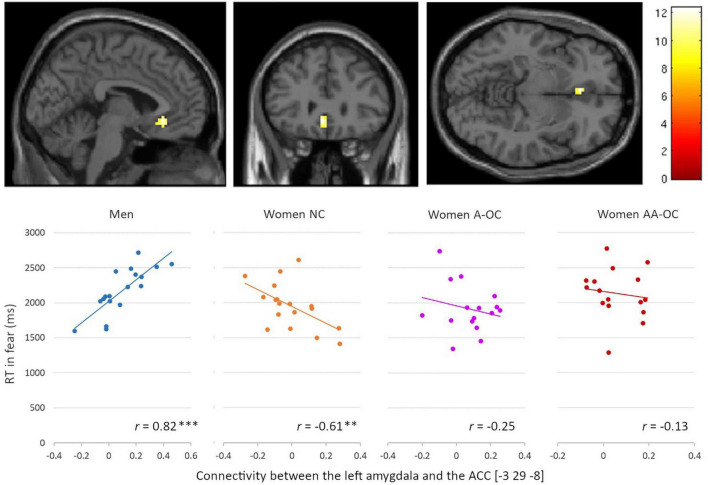
Relationship between the left amygdala-anterior cingulate cortex connectivity and reaction time (RT) for fearful faces by group. Connectivity strength and RT were found to be positively correlated for men, whereas they were negatively correlated for naturally cycling women. **p < 0.01, ***p < 0.001.

For the emotion of fear, we observed a significant group*RT interaction for the connectivity between the left amygdala and the left anterior cingulate cortex (ACC) [−3 29 −8], *k* = 18 voxels, *F* = 12.35, p_FWE_ = 0.02. Connectivity strength and RT were found to be positively correlated for men (*r*_17_ = 0.82), whereas it was negatively correlated for naturally cycling women (*r*_16_ = −0.61) (Figure 5). The lower the left amygdala-ACC connectivity strength, the faster fear recognition in men, while the slower fear recognition in naturally cycling women.

**FIGURE 6:**
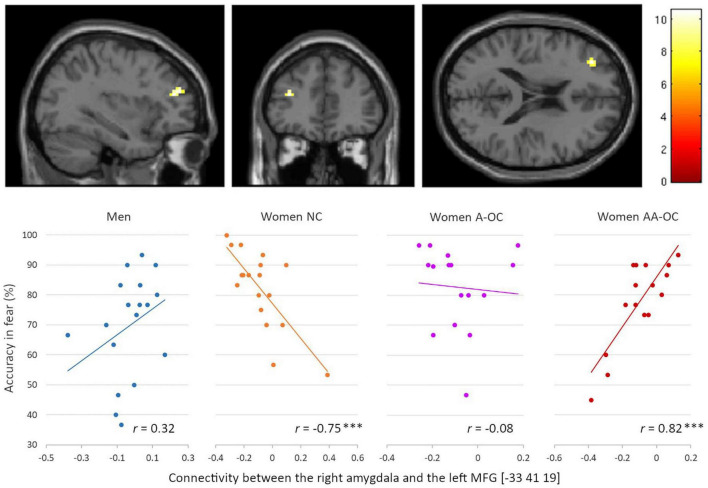
Relationship between the right amygdala-left middle frontal gyrus connectivity and accuracy for fearful faces by group. Connectivity strength and accuracy were found to be negatively correlated for naturally cycling women, whereas they were positively correlated for women on anti-androgenic oral contraceptives. ***p < 0.001.

Also for the emotion of fear, we observed a significant group*accuracy interaction for the connectivity between the right amygdala and the left middle frontal gyrus (MFG) [−33 41 19], *k* = 15 voxels, *F* = 10.61, p_FWE_ = 0.04. Connectivity strength and accuracy were found to be negatively correlated for naturally cycling women (*r*_16_ = −0.75), whereas it was positively correlated for women on AA-OC (*r*_14_ = 0.82) (Figure 6). The lower the right amygdala-left MFG connectivity strength, the higher fear recognition accuracy in naturally cycling women, while the lower fear recognition accuracy in women using AA-OC.

**FIGURE 7:**
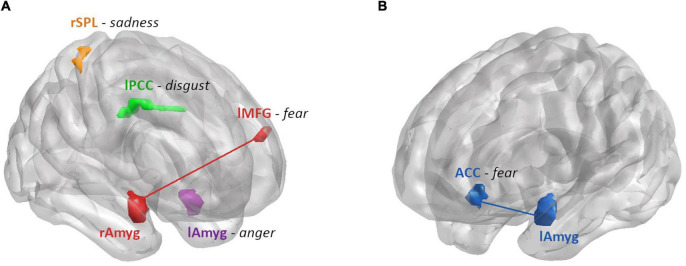
Summary of the brain areas that showed a differential relationship between ALFF or connectivity strength and emotion recognition performance by hormonal status in women **(A)** and sex **(B)**. **(A)** Areas modulated by hormonal status: ALFF and RT for anger were found to be moderately positively correlated for women on A-OC in the left amygdala (purple). ALFF and accuracy for disgust were found to be moderately negatively correlated for women on AA-OC in the left PCC (green). ALFF and accuracy for sadness were found to be positively correlated for naturally cycling women, whereas they were negatively correlated for women on AA-OC in the right SPL (orange). Connectivity strength between right amygdala-left MFG (red) and accuracy for fear were found to be negatively correlated for naturally cycling women, whereas they were positively correlated for women on AA-OC. **(B)** Areas modulated by sex: Connectivity strength between the left amygdala-ACC (blue) and RT for fear were found to be positively correlated for men, whereas they were negatively correlated for naturally cycling women.

In summary, we found the ALFF in the left amygdala, the left PCC and the right SPL related to anger, disgust and sadness recognition performance (respectively), depending on hormonal status in women (Figure 7). Regarding the connectivity from the amygdalae, the connectivity strength to ACC and left MFG was related to fear recognition performance, depending on sex and hormonal status (Figure 7).

## Discussion

The current study set out to investigate whether hormonal contraceptive effects on emotion recognition performance were related to gray matter volume, oscillatory activity and connectivity of emotion-related brain areas at rest. Notably, no behavioral differences according to sex, menstrual cycle phase or hormonal contraceptive use emerged. These results are in accordance with previous studies who did not find a link between emotion recognition performance and hormonal status or pill use (Sundström Poromaa and Gingnell, 2014; Radke and Derntl, 2016; Shirazi et al., 2020), but differ from other studies who did find an effect (Conway et al., 2007; Derntl et al., 2008a; Hamstra et al., 2014; Pahnke et al., 2019; Gurvich et al., 2020). Habituation over sessions could be a possible explanation for the lack of differences between different genders and pill type. Participants recognized anger, fear and disgust faster during the second and third session and were more accurate in their identification of sadness during the second and third session compared to the first session, which could explain the similar emotion recognition performance in the combined data.

Regarding the question, whether certain characteristics of the resting brain were predictive of participants ability to recognize five basic emotions (anger, fear, sadness, happiness, disgust), while controlling for participant’s sex and hormonal status, no general pattern of emotion recognition predictability emerged. Instead, results were strongly dependent on (a) the respective emotion and (b) the hormonal status of participants. Thus, contrary to our predictions, resting brain characteristics did not mediate oral contraceptive effects on performance, but oral contraceptive use emerged as a moderator of brain-behavioral associations. This suggests that depending on participants’ hormonal status, certain brain areas tune to the recognition of specific emotions. In the following, we will first discuss the differences that emerged between men and naturally cycling women, and then discuss the differences between naturally cycling women and OC users.

Sex differences did emerge in the prediction of emotion recognition performance by the left amygdala amplitude of low frequency oscillations (ALFF) for anger, as well as left and right amygdala connectivity for fear. The recognition of anger was faster in men with higher ALFF, but slower in naturally cycling women with higher ALFF. This suggests that a stronger oscillatory activity of the amygdala at rest facilitates the recognition of anger in men, but impairs the recognition of anger in naturally cycling women. Repple et al. (2018) show that men have a stronger response in the amygdala after provocation, and that this response correlated with trait anger. They also showed a positive association between the anterior cingulate cortex activity when provoked and a more aggressive response.

The connectivity between the amygdala and anterior cingulate cortex (ACC) is involved in aversive learning and important for threatening stimuli processing (Klavir et al., 2013). Inhibitory functional coupling during threatening stimuli processing has been shown (Toyoda et al., 2011) and in rodents inactivation of the ACC inputs to the amygdala lead to an enhanced fear response (Jhang et al., 2018). Projections from the ACC might control anxiety in threatening situations (Jhang et al., 2018), explaining the differential connectivity between the amygdala and ACC found in this manuscript. The more strongly the left amygdala recruits the ACC, the slower men are in recognizing fear, but the faster are naturally cycling women. These results suggest that a stronger connectivity of the amygdala at rest impairs the recognition of fear in men, but facilitates the recognition of fear in women. An interesting finding of Kogler et al. (2016) shows that cortisol levels are negatively associated with resting state functional connectivity of the amygdala with the ACC in women, and positively associated in men. Higher levels of cortisol can lower connectivity in women, hence making their response slower, while the opposite occurs for men with high cortisol levels. This is also of interest in the light of the findings of Bouma et al. (2009) who showed reduced cortisol reaction following hormonal contraceptive use.

Apart from that, the coupling of the amygdala and ACC during face processing shifts from positive to negative over age (Kujawa et al., 2016). Young people display greater ACC activation to emotional faces due to inhibitory effects on amygdala activation (Kujawa et al., 2016). Although in the present sample, brain behavior association were not modulated by age and this does not explain the faster recognition by women, where the opposite effect was found, it could be that such an effect appears more strongly in adult men than women. Another possible explanation is that women use the connection between the ACC and the amygdala to recognize fear faster, because a fearful face is indicative of a threat and elicits a fear response. Rahman et al. (2004) reported a faster response to facial emotional stimuli for women compared to men. They suggest that the faster response in women is a by-product of face recognition, which has a faster response to non-congruent gender faces than men do.

In addition, the more strongly the right amygdala recruits the left middle frontal gyrus (MFG), the more accurate men are in recognizing fear, but the worse naturally cycling women recognize fear. Studies have shown that the MFG is involved in fearful engagement (Thielscher and Pessoa, 2007), and that signaling from the middle frontal gyrus to the amygdala is suppressed in response to emotional distractors (Yamasaki et al., 2002). Since the MFG inhibits the right amygdala, it is possible that the less inhibition there is of the amygdala, the better men recognize fear.

Regarding OC effects the pattern of results was opposite as expected. While we hypothesized a more masculinized pattern of brain-behavior associations in androgenic OC users, but a feminized pattern in anti-androgenic OC users, the opposite pattern emerged. Anti-androgenic OC-users stood out in that they showed strong brain-behavior associations, usually in the opposite direction as naturally cycling women, while androgenic OC-users showed a pattern similar to, but weaker, than naturally cycling women. This was observed in (i) the ALFF of the left amygdala, (ii) the ALFF of the posterior cingulate gyrus (PCC), (iii) the ALFF of the superior parietal lobe (SPL), and (iv) the connectivity between right amygdala and left MFG.

For most of the sample, higher ALFF in the PCC and SPL was associated with higher accuracy in recognizing disgust and sadness respectively. On the contrary, in anti-androgenic OC-users, emotion recognition accuracy dropped with higher ALFF in these areas. Regarding the left amygdala, anti-androgenic OC users showed a similar increase in anger recognition speed as men with higher oscillatory activity. This is of interest in relation to possible effects of hormonal contraceptive use on adverse mood symptoms and related disorders (Cheslack-Postava et al., 2015; Skovlund et al., 2016; de Wit et al., 2020). The cingulate cortex is known to play a role in emotional processing (Vogt, 2005), and activity in its posterior section is higher when observing disgusting stimuli compared to neutral stimuli (Benuzzi et al., 2008). Activation in PCC in a face-related has been related to both estradiol and testosterone levels in NC women (Rupp et al., 2009), which may explain its modulation by OCs with estrogenic, but anti-androgenic activity. As for the SPL, previous research has shown that the display of sadness leads to increased activity in the left SPL (McLellan et al., 2012). Functional resting-state connectivity between the right basolateral amygdala and the SPL are associated with the personality trait sadness (Deris et al., 2017). Finally, regarding the connectivity of the right amygdala and left MFG, the MFG seems to suppress emotional stimuli (Yamasaki et al., 2002) and inhibits the right amygdala, explaining the better fear recognition in anti-androgenic oral contraceptives.

While these results are somewhat surprising given that masculinizing effects were expected in androgenic OC users due to the binding affinity of androgenic progestins to the androgen receptor, there are two possible explanations for this pattern. First, multiple mechanisms might facilitate androgenic actions in OC users, some of which are also present in anti-androgenic OC users. For instance, OC-use reduces the levels of progesterone, which has a higher affinity for the enzyme 5α-dehydrogenase compared to testosterone (Wright et al., 1983). Thus, in OC-users more testosterone can be converted into the physiologically more active dihydrotestosterone, which has a higher binding affinity for the androgen receptor. Second, it is possible that these differences between groups are not due to activational effects of sex hormones in adulthood, but the result of organizational effects of sex hormones. Anti-androgenic progestins are often prescribed in women who present with at least slight androgenic symptoms, e.g., acne or hirsutism (Fuchs et al., 2019). It is thus possible that our groups were subject to a selection bias and women in the group of anti-androgenic OC-users either had higher androgen levels until they started taking their contraceptive or have a higher sensitivity to androgens.

Despite the extended use of OCs, only few studies have investigated their effects on brain activity and connectivity of emotion-related brain regions. Relatedly, the impact of OCs use on psychological well-being and emotional regulation, and a mechanistic approach on how this effects may be exerted is lacking. Understanding the hormonal contraceptive effects on emotion recognition performance related to brain activity and connectivity at rest provide some groundwork for future studies. In the present study, we showed that the oscillatory activity in emotion-related brain areas, such as PCC, SPL and amygdala were indeed predictive of participant’s ability to recognize facial emotional expressions, particularly the emotions anger, disgust, fear and sadness (Figure 7). Furthermore, these results were dependent on the use and type of COCs. Amygdalae connectivity were also predictive of fear recognition performance.

## Data Availability Statement

Data and scripts are openly available online at http://webapps.ccns.sbg.ac.at/OpenData/. MR images are available upon request from the corresponding author.

## Ethics Statement

The studies involving human participants were reviewed and approved by Ethics Committee of the University of Salzburg. The patients/participants provided their written informed consent to participate in this study. Written informed consent was obtained from the individual(s) for the publication of any potentially identifiable images or data included in this article.

## Author Contributions

BP designed the study together with HK, acquired the data and supervised the data analysis and manuscript preparation. SM-H and EH-L performed the data analysis and wrote the first draft of the manuscript. MK and MA provided input on the data analysis. All authors read and approved the final manuscript.

## Conflict of Interest

The authors declare that the research was conducted in the absence of any commercial or financial relationships that could be construed as a potential conflict of interest.

## Publisher’s Note

All claims expressed in this article are solely those of the authors and do not necessarily represent those of their affiliated organizations, or those of the publisher, the editors and the reviewers. Any product that may be evaluated in this article, or claim that may be made by its manufacturer, is not guaranteed or endorsed by the publisher.

## Footnotes

1
http://faces.mpib-berlin.mpg.de/

2
https://www.R-project.org/

3
http://dbm.neuro.uni-jena.de/vbm/

4
http://www.fil.ion.ucl.ac.uk/spm/

5
https://afni.nimh.nih.gov/

6
http://www0.cs.ucl.ac.uk/staff/gridgway/vbm/get_totals.m

7
http://www.nitrc.org/projects/conn
